# Antihypertensive Treatment Patterns in CKD Stages 3 and 4: The CKD-REIN Cohort Study

**DOI:** 10.1016/j.xkme.2024.100912

**Published:** 2024-10-09

**Authors:** Margaux Costes-Albrespic, Sophie Liabeuf, Solène Laville, Christian Jacquelinet, Christian Combe, Denis Fouque, Maurice Laville, Luc Frimat, Roberto Pecoits-Filho, Oriane Lambert, Ziad A. Massy, Bénédicte Sautenet, Natalia Alencar de Pinho, CKD-REIN study group, Natalia Alencar de Pinho, Christian Combe, Denis Fouque, Luc Frimat, Aghilès Hamroun, Christian Jacquelinet, Maurice Laville, Sophie Liabeuf, Ziad A. Massy, Abdou Omorou, Christophe Pascal, Roberto Pecoits-Filho, Bénédicte Stengel, Céline Lange, Oriane Lambert, Marie Metzger

**Affiliations:** 1 Centre for Research in Epidemiology and Population Health, Paris-Saclay University, Inserm U1018, Versailles Saint-Quentin University, Clinical Epidemiology Team, Villejuif, France; 2 Pharmaco-epidemiology Unit, Department of Clinical Pharmacology, Amiens-Picardie University Medical Center, Amiens, France; 3 MP3CV Laboratory, Jules Verne University of Picardie, Amiens, France; 4 Biomedicine Agency, La Plaine Saint-Denis, France; 5 Nephrology, Transplantation, Dialysis, and Apheresis Department, University Hospital of Bordeaux, Bordeaux University, Bordeaux, France; 6 Inserm U1026, Bordeaux University, Bordeaux, France; 7 Nephrology Department, Lyon-Sud University Hospital, Claude Bernard University Lyon 1, Pierre-Bénite, France; 8 Carmen INSERM U1060, Claude Bernard University Lyon 1, Pierre-Bénite, France; 9 Nephrology Department, University Regional Hospital of Nancy, Vandoeuvre-lès-Nancy, France; 10 APEMAC, Lorraine University, Nancy, France; 11 Arbor Research Collaborative for Health, Ann Arbor MI, USA; 12 Nephrology Department, Ambroise Paré University Hospital, APHP, Boulogne-Billancourt, Paris, France; 13 Nephrology, Arterial Hypertension, Dialysis, and Renal Transplantation Department, INSERM U1246 SPHERE, Nantes, France

**Keywords:** chronic kidney disease, hypertension, blood pressure, antihypertensive agents, renin-angiotensin system

## Abstract

**Rationale & Objective:**

Blood pressure (BP) control is essential for preventing cardiorenal complications in chronic kidney disease (CKD), but most patients fail to reach BP target. We assessed longitudinal patterns of antihypertensive drug prescription and systolic BP.

**Study Design:**

Prospective observational cohort study.

**Setting & Population:**

In total, 2,755 hypertensive patients with CKD stages 3-4, receiving care from a nephrologist, from the French CKD–Renal Epidemiology and Information Network (CKD-REIN cohort study).

**Exposure:**

Patient factors, including sociodemographic characteristics, medical history, and laboratory data, and provider factors, including number of primary care physician and specialist encounters.

**Outcomes:**

Changes in antihypertensive drug-class prescription during follow-up: add-on or withdrawal.

**Analytical Approach:**

Hierarchical shared-frailty models to estimate hazard ratios (HR) to deal with clustering at the nephrologist level and linear mixed models to describe systolic BP trajectory.

**Results:**

At baseline, median age was 69 years, and mean estimated glomerular filtration rate was 33 mL/min/1.73 m². In total, 66% of patients were men, 81% had BP ≥ 130/80 mm Hg, and 75% were prescribed ≥2 antihypertensive drugs. During a median 5-year follow-up, the rate of changes of antihypertensive prescription was 50 per 100 person-years, 23 per 100 for add-ons, and 25 per 100 for withdrawals. After adjusting for risk factors, systolic BP, and the number of antihypertensive drugs, poor medication adherence was associated with increased HR for add-on (1.35, 95% confidence interval [CI], 1.01-1.80), whereas a lower education level was associated with increased HR for withdrawal (1.23, 95% CI, 1.02-1.49) for 9-11 years versus ≥12 years. More frequent nephrologist visits (≥4 vs none) were associated with higher HRs of add-on and withdrawal (1.52, 95% CI, 1.06-2.18; 1.57, 95% CI, 1.12-2.19, respectively), whereas associations with visit frequency to other physicians varied with their specialty. Mean systolic BP decreased by 4 mm Hg following drug add-on but tended to increase thereafter.

**Limitations:**

Lack of information on prescriber and drug dosing.

**Conclusions:**

In patients with CKD and poor BP control, changes in antihypertensive drug prescriptions are common and relate to clinician preferences and patients’ tolerability. Sustainable reduction in systolic BP after add-on of a drug class is infrequently achieved.

## Plain-Language Summary

Blood pressure (BP) control remains unattained in most patients with chronic kidney disease (CKD), raising questions about how antihypertensive treatment is managed. Our study highlights dynamic, yet heterogeneous patterns of antihypertensive drug prescriptions in patients with CKD stages 3-4 receiving care from a nephrologist over 5 years of follow-up. Modifiable factors such as high body mass index and poor medication adherence were associated with higher hazard of adding-on an antihypertensive drug class, independently of baseline BP and antihypertensive treatment. Similarly, lower education level was associated with antihypertensive drug withdrawn, as was more frequent visits to primary care physicians, underlining the importance of coordinated care. Sustainable reduction in systolic BP after add-on of a drug class is infrequently achieved and may be related to drug withdrawal and poor treatment adherence.

Hypertension is the leading modifiable risk factor for premature death, affecting 1.4 billion people worldwide.^1^ A possible cause and consequence of chronic kidney disease (CKD), hypertension is also its most common comorbid condition.^2^^,^^3^ Strict blood pressure (BP) control unambiguously improves survival and cardiovascular (CV) outcomes in this population.^4^, ^5^, ^6^, ^7^ Guidelines recommend systolic BP levels from 130-139 to <120 mm Hg in CKD,^8^, ^9^, ^10^ but these targets are not met by many patients, with considerable international variation.^11^

Achieving adequate BP control most often requires associating 2 or more antihypertensive drug classes.^12^^,^^13^ Renin-angiotensin system (RAS) inhibitors are recommended as first-line treatment in patients with CKD, more effective than active controls in improving kidney and CV outcomes.^8^, ^9^, ^10^ Absent other compelling indications or contraindications, calcium-channel blockers (CCBs) and diuretics are interchangeably recommended as second- and third-line drugs, but evidence for preferring one or the other lacks consistency.^9^^,^^10^^,^^14^, ^15^, ^16^ The management of hypertension in patients with CKD is complex because of their multiple comorbid conditions and prescriptions for which different drug classes may be contraindicated or poorly tolerated, and prescribers may have preferences.^17^

In a nationally representative CKD cohort with poor BP control, we hypothesized that antihypertensive treatment is rarely reassessed. We comprehensively analyzed rates of changes in antihypertensive drug prescription in CKD (add-on and withdrawal), the association of these changes with patient- and provider-related factors, and the short-term systolic BP trajectory in patients with and without an add-on of an antihypertensive drug over follow-up.

## Methods

### Data Source and Population

The CKD–Renal Epidemiology and Information Network (CKD-REIN) is a prospective cohort study conducted in 40 French nephrology clinics, nationally representative (geographically and by legal status). From 2013-2016, we included 3,033 patients with moderate-to-advanced CKD not transplanted nor on maintenance dialysis. The CKD-REIN cohort study's complete rationale, design, and methods are available elsewhere.^18^ The French National Institute of Health and Medical Research's institutional review board approved the protocol (reference: IRB00003888); the study was registered at ClinicalTrials.gov (NCT03381950). All study participants were aged ≥18 years and provided informed consent.

We identified patients with a diagnosis of arterial hypertension in medical records (n = 2,605), a prescription of antihypertensive drugs (n = 2,822), or a systolic/diastolic BP ≥130/80 mm Hg at least twice (n = 2,036), ie, a total of 2,957 patients at baseline. After excluding patients with missing data for nephrologist identification (n = 2), BP at baseline (n = 14), or antihypertensive drug status over follow-up (n = 186), we studied 2,755 patients (Fig S1).

### Explanatory Variables

Baseline and annual data collected from interviews, records, and self-administered questionnaires included sociodemographic characteristics, medical history, and laboratory data. Diabetes was defined by glucose-lowering drug prescription(s), glycated hemoglobin (HbA1c) ≥6.5%, fasting glucose ≥7 mmol/L, or nonfasting glucose ≥11 mmol/L. CV history at baseline included heart failure (HF), coronary artery disease (CAD), cerebrovascular disease, peripheral arterial disease, and dysrhythmias. Spot or 24-hour urine tests, prescribed as routine care, were used to calculate sodium-creatinine ratios.^19^ Urinary albumin-creatinine ratios (ACR) were measured or estimated from protein-creatinine ratios.^20^ The glomerular filtration rate (eGFR) was estimated with the 2021 Chronic Kidney Disease Epidemiology Collaboration (CKD-EPI) equation.^21^ CKD is classified by eGFR (G1-G5, with cutoffs at 90, 60, 30, and 15 mL/min/1.73 m^2^) and ACR (A1-A3, with cutoffs at 30 and 300 mg/g). Height and weight were measured by nephrologists or outpatient nurses during a routine visit, and used for body mass index (BMI, kg/m^2^) calculation. Adherence to medications was assessed with the Girerd score,^22^ calculated with 6 items and described in 3 categories, good, moderate, and poor. Participants reported the number of visits to their primary care physician (PCP) and specialists in the year preceding enrollment through self-administered questionnaires.

### Information on BP and Antihypertensive Drugs

Study protocol required BP to be measured at least twice, in sitting position, after a 5-minute rest; the average of these measurements was used in all analysis. These measurements were performed by nephrologists or outpatient nurses once a year by protocol or more frequently during routine visits. Participants were asked to bring all drug prescriptions (from any doctor) over the past 3 months to their inclusion visit and all prescriptions for the preceding year to each annual follow-up visit. Antihypertensive drugs were then coded according to the international Anatomic Therapeutic and Chemical thesaurus (Table S1) and analyzed at levels 3 (pharmacologic subgroup) and 4 (chemical subgroup) of the Anatomic Therapeutic and Chemical hierarchy.^23^

We evaluated changes in antihypertensive drug classes prescribed during follow-up: add-on, switch, and withdrawal. Patients were followed up until initiation of kidney replacement therapy (KRT), death before KRT, completion of 5 years of follow-up, loss to follow-up, or censoring on December 31, 2020.

### Statistical Analysis

Baseline characteristics and antihypertensive drug classes were described overall and by subgroups at baseline (systolic BP, CKD G stage, and CKD A stage). All changes were considered to estimate overall rates of add-ons, withdrawals, and switches. All patients were considered at risk of any change and any add-on during their follow-up, only periods in which patients were prescribed at least one antihypertensive drug were considered for any withdrawal. For specific drug classes, periods when patients were not prescribed the drug were considered for add-on rate, and periods when patients were prescribed the drug were considered for withdrawal rate. Because few switches were identified during follow-up, we estimated class-specific rates only for add-ons or withdrawals. The first 3 changes in the number of classes during follow-up were depicted graphically with a Sankey plot.

We estimated crude and adjusted cause-specific hazard ratios (HRs) and 95% confidence intervals (95% CIs) of an antihypertensive drug class add-on or withdrawal (first event) associated with patient- and provider-related factors using hierarchical shared-frailty models. Models accounted for clustering at the nephrologist level through a lognormal random effect. Factors previously identified in the literature as risk factors for hypertension (eg, comorbid conditions) and factors describing provider or health care features (eg, number of medical visits) were modeled as fixed effects (details in Item S1: Supplementary Methods). We tested the interaction term between systolic BP and the number of antihypertensive drug classes, which was significant for add-on. Log-linearity and proportional hazard assumptions were checked graphically with Martingale and Schoenfeld residuals, respectively. Death and KRT initiation were competing risks.

To describe short-term trends in systolic BP, we performed linear mixed models with random intercept and slope for patients with and without add-on of an antihypertensive drug class. Fixed effects were time, add-on status, and the interaction between these 2. Time zero corresponds to the timing of add-on and 8.4 months from baseline (median time to first add-on) for patients with and without add-on, respectively. We performed multiple imputation of missing data with a multivariate normal model fitted using a Markov Chain Monte Carlo method that takes a 2-level data structure into account (JOMO package for R software).^24^ We used Rubin and Schencker’s framework to combine estimates across 30 imputed datasets.^25^ As a sensitivity analysis, we added the urinary sodium-creatinine ratio (both in mmol/L) in patients with complete data (n = 1,975). Statistical significance was defined by *P* < 0.05. Statistical analyses were performed with SAS software, version 9.4 (SAS Institute Inc) and R, version 3.6.2 (R Foundation for Statistical Computing).

## Results

Among 2,755 patients, median age was 69 years, 933 (34%) were women, and the mean eGFR was 33 mL/min/1.73 m2. At baseline 2,232 patients (81%) had BP ≥ 130/80, and 1,515 (55%) a mean BP ≥140/90 mm Hg. Two or more antihypertensive drug classes were prescribed to 2,066 (75%). Those with a lower systolic BP were younger; more highly educated; had less severe albuminuria; and had lower frequencies of CAD, HF, or cerebrovascular disease (Table 1). Most patients received care in clinics at university hospitals, especially patients in the lowest systolic BP group (Table S2). More than half the patients (55%) had seen a PCP at least 4 times in the year before study enrollment, 2,327 (97%) had seen the nephrologist at least once, and 1,692 (70%) a cardiologist or a diabetologist.

**Table 1:** Patient Characteristics at Baseline, Overall and by Systolic Blood Pressure Level (mm Hg)

Systolic Blood Pressure Levels Baseline Characteristics	All (n = 2,755)	<120 (n = 282)	120-129 (n = 415)	130-139 (n = 615)	140-159 (n = 946)	≥ 160 (n = 497)	Missing Data N (%)
**Age (y), median (IQR)**	69 (61-77)	65 (51-73)	66 (56-72)	68 (59-76)	70 (64-77)	71 (66-78)	0 (0%)
**Women, n (%)**	933 (34%)	107 (38%)	145 (35%)	205 (33%)	308 (33%)	168 (34%)	0 (0%)
**Education (years), n (%)**							32 (1.2%)
<9	413 (15%)	37 (13%)	51 (12%)	89 (15%)	146 (16%)	90 (18%)	
9-11	1,356 (50%)	125 (45%)	184 (45%)	291 (48%)	493 (52%)	263 (54%)	
≥12	954 (35%)	114 (42%)	175 (43%)	231 (37%)	296 (32%)	138 (28%)	
**Adherence**^a^							0 (0%)
Good	1,029 (37%)	106 (38%)	154 (37%)	244 (40%)	353 (37%)	172 (35%)	
Moderate	1,527 (55%)	153 (54%)	235 (57%)	322 (52%)	527 (56%)	290 (58%)	
Poor	199 (8%)	23 (8%)	26 (6%)	49 (8%)	66 (7%)	35 (7%)	
**eGFR (mL/min/1.73** **m**^**2**^**), mean (SD)**	33 (12)	34 (12)	34 (12)	33 (12)	33 (12)	32 (12)	0 (0%)
**Albumin-creatinine ratio (mg/g), median (IQR)**	122 (25-544)	71 (16-402)	81 (22-388)	100 (23-390)	134 (24-615)	244 (39-824)	397 (14%)
**Medical history n (%)**							
Diabetes	1,202 (44%)	101 (36%)	150 (36%)	241 (39%)	460 (49%)	250 (51%)	6 (0.2%)
Any cardiovascular disease	1,466 (54%)	152 (54%)	201 (49%)	305 (50%)	522 (56%)	286 (58%)	34 (1.2%)
Coronary artery disease (CAD)	680 (25%)	75 (27%)	97 (24%)	130 (22%)	251 (27%)	127 (26%)	51 (1.9%)
Heart failure (HF)	368 (13%)	65 (23%)	46 (11%)	82 (13%)	119 (13%)	56 (11%)	6 (0.2%)
Cerebrovascular disease (CVD)	311 (12%)	26 (9%)	46 (11%)	65 (11%)	105 (11%)	69 (14%)	63 (2.3%)
Acute kidney injury	593 (22%)	66 (24%)	90 (22%)	125 (20%)	199 (21%)	113 (23%)	32 (1.2%)
**BMI (kg/m**^**2**^**), mean (SD)**	28.8 (5.8)	27.6 (5.9)	28.1 (5.6)	28.4 (5.6)	29.5 (5.9)	29.4 (6.0)	57 (2.1%)
**Systolic BP (mm Hg), mean (SD)**	142 (20)	112 (7)	124 (3)	134 (3)	148 (6)	172 (12)	0 (0%)
**Diastolic BP (mm Hg), mean (SD)**	78 (12)	68 (9)	74 (9)	77 (9)	79 (11)	85 (13)	1 (<0.1%)
**Number of BP measurements over the follow-up, median (IQR)**	7 (5 - 11)	7 (5 - 10)	7 (4 - 10)	8 (5 - 11)	7 (5 - 10)	8 (5 -11)	0 (0%)
**Time interval between BP measurements (mo), median (IQR)**	5.4 (3.2-7.1)	5.8 (3.9-8.2)	5.8 (3.5-8.3)	5.7 (3.5-7.5)	5.1 (3.0-6.9)	4.8 (3.0-6.4)	0 (0%)
**Urinary sodium-creatinine ratio, median (IQR)**	12 (9-17)	11 (8-15)	11 (8-16)	12 (8-16)	12 (9-17)	13 (10-18)	780 (28%)
**Number of drugs prescribed, median (IQR)**	8 (5-11)	7.5 (5-10)	7 (5-10)	7 (5-10)	8 (6-11)	8 (6-11)	0 (0%)
**Number of antihypertensive drugs prescribed, median (IQR)**	2 (1-3)	2 (1-3)	2 (1-3)	2 (1-3)	3 (2-4)	3 (2-4)	0 (0%)

Abbreviations: BMI, body mass index; BP, blood pressure; CAD, coronary artery disease; CVD, cerebrovascular disease; eGFR, estimated glomerular filtration rate; HF, heart failure; IQR, interquartile range; SD, standard deviation.

a Adherence to medications was assessed with the Girerd score, calculated with 6 items and described in 3 categories, good, moderate, and poor.^22^

### Antihypertensive Drug Prescriptions at Baseline

The most frequently prescribed antihypertensive drug classes were RAS inhibitors (RASis) (2,133 [77%]), diuretics (1,531 [56%]), CCBs (1,308 [48%]), and β-blockers (1,170 [42%]) (Table S3). Amiloride, methyldopa, pyrimidine derivatives, and rauwolfia alkaloids (reserpine) were rarely or never described (≤1%). Overall, 257 antihypertensive drug regimens were observed at baseline (10 single class and 247 class combinations, Table S4). The top 5 prescriptions included RASi alone (405 [15%]) or in combination (1,735 [63%]), followed by diuretics + RASi (246 [9%]), diuretics + CCBs + RASi (241 [9%]), diuretics + β-blockers + RASi (229 [8%]), and diuretics + β-blockers + CCBs + RASi (226 [8%]). No other antihypertensive drug regimen was prescribed to more than 6% of patients.

### Rates of Changes in Antihypertensive Drug-Class Prescriptions

**Figure 1:**
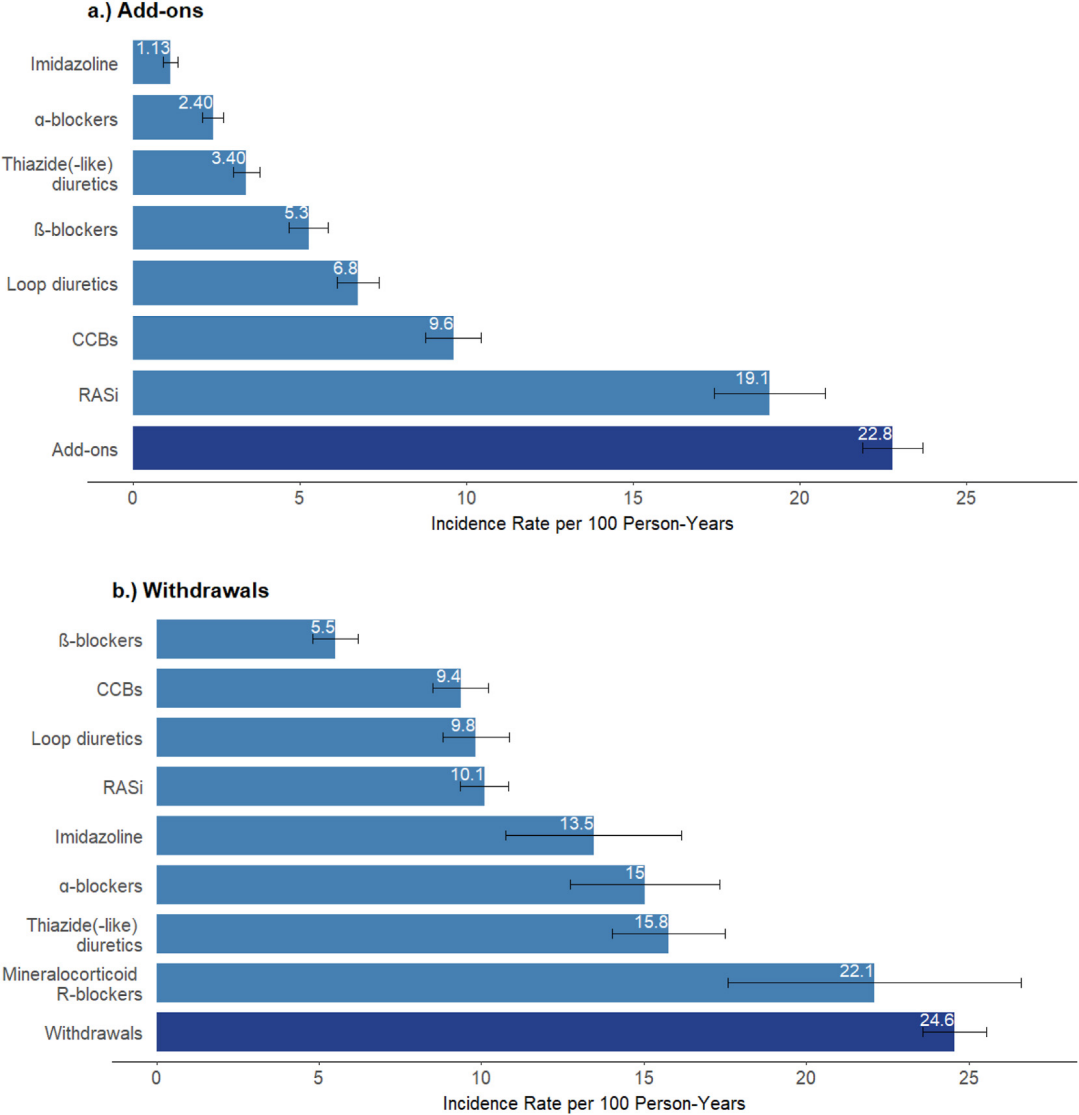
Rates of add-ons and withdrawals (with 95% confidence intervals) by antihypertensive drug class. Figure displays add-on and withdrawal rates for drug classes with at least 85 events. Rates for drug classes with fewer events, as well as number of events and persons at risk for all drug classes are shown in Supplementary Table 5.

During a median 5-year follow-up (interquartile range [IQR], 4.6-5.2), we observed 2,411 drug-class add-ons, 2,463 withdrawals, and 391 switches. The number of add-ons and withdrawals was highest for RASi (507 and 725, respectively) and CCBs (514 and 449). The rate of any change of antihypertensive prescription was 50 per 100 person-years (PY) (Table S5). The rates of add-on and withdrawal of an antihypertensive drug class were nearly the same (23 and 25 per 100 PY, respectively); the switch rate was much lower at 3.90 per 100 PY. Add-on rates were highest for RASi (19 per 100 PY) and CCBs (9.6 per 100 PY), and withdrawal rates were highest for mineralocorticoid receptor antagonists (MRA) (22 per 100 PY) and thiazide diuretics (16 per 100 PY, Fig 1, Table S5).

**Figure 2:**
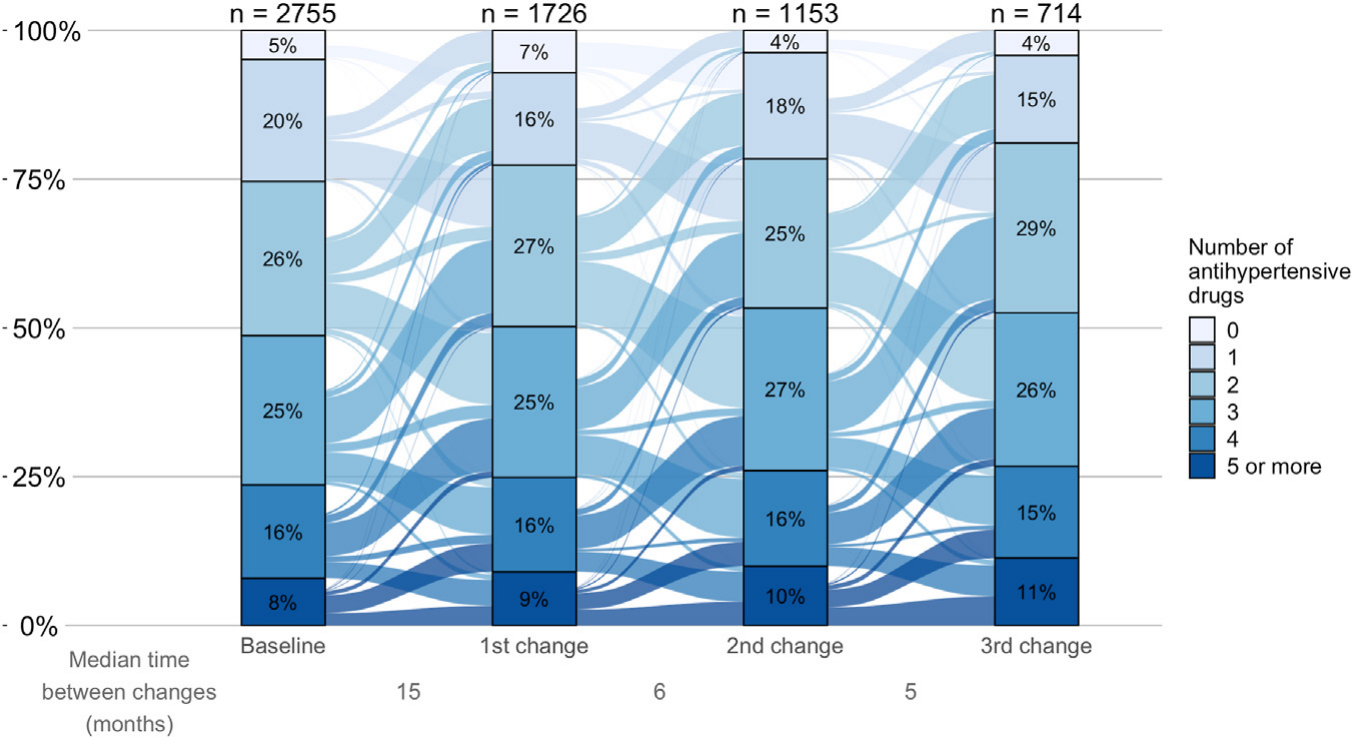
Changes in the number of antihypertensive drugs prescribed during follow-up. In this Sankey plot, bars represent the distribution of the number of antihypertensive drug classes at baseline and at the first 3 change time points. X-axis depicts the median time to each prescription change (in months). Y-axis represents the percentage of patients with a given number of drug classes prescribed. Links from one bar to another are proportional to the flow rate of patients whose number of antihypertensive drug classes changed.

At the time of the add-on, the median number of antihypertensive drug classes prescribed was higher among patients who were added MRAs, α-blockers, or imidazoline (median of 3; IQR, 2-4, Table S6) than for patients who were added other classes (median of 2; IQR, 1-3). These patients were less often prescribed RAS inhibitors (59%-71% at the time of add-on of a drug class, RAS inhibitors excluded) compared with the overall sample (77% at baseline). The median number of antihypertensive drug classes prescribed at the time of prescription withdrawal was 5 (IQR, 4-5) for imidazoline, 4 (IQR, 3-5) for MRAs, and α-blockers, and 3 (IQR, 2-4) for other drug classes. Because almost the same proportion of patients had a drug class added as withdrawn during follow-up, the distribution of the number of antihypertensive drug classes prescribed remained virtually unchanged over time (Fig 2).

### Factors Associated with Changes of Antihypertensive Drug Prescriptions

For patients prescribed up to 2 antihypertensive drug classes, the hazard of adding an antihypertensive drug class was higher at higher levels of baseline systolic BP, starting from values around 140 mm Hg (Table 2, Supplementary Table S7, and Fig S2). Inversely, the higher the baseline number of these classes, the lower the hazard of adding another. After adjusting for these and other clinical and health care-related characteristics, 3 groups of patients—older, with higher BMI, and with poor adherence—had a higher hazard of an add-on. Patients with more visits to the nephrologist or to another specialist and care in a private nonprofit nephrology facility versus a nonuniversity hospital also had a higher hazard of an add-on.

**Table 2:** Adjusted Hazard Ratios For The Add-On and Withdrawal of an Antihypertensive Drug Class Associated with Patient- and Provider-Related Factors

Factors	HR (95% CI) Adjusted Models	
	Add-ons^a^	Withdrawals
**Systolic BP (mm Hg, reference: 120** **mm Hg**^b^**)**		
110 mm Hg	1.00 (0.80-1.25)	1.13 (0.99-1.30)
130 mm Hg	1.03 (0.91-1.17)	0.93 (0.86-1.00)
140 mm Hg	1.11 (0.97-1.28)	0.91 (0.83-0.99)
150 mm Hg	1.21 (1.03-1.42)^c^	0.90 (0.80-1.01)
**Age (per 10-y increase)**	1.09 (1.01-1.17)^c^	1.06 (0.99-1.14)
**Men**	0.95 (0.81-1.12)	0.99 (0.85-1.16)
**Education (years, reference: ≥12** **y)**		
9-11	1.15 (0.90-1.46)	1.23 (1.02-1.49)^c^
<9	1.08 (0.91-1.28)	0.96 (0.82-1.13)
**Adherence (reference: good)**		
Moderate	1.13 (0.97-1.32)	1.14 (0.98-1.34)
Poor	1.35 (1.01-1.80)^c^	0.95 (0.70-1.29)
**Diabetes**	0.99 (0.83-1.19)	0.92 (0.78-1.09)
**eGFR (per decrease of 10** **mL/min/1.73** **m**^**2**^**)**	1.01 (0.94-1.08)	1.05 (0.98-1.12)
**ACR (mg/g, per increase of 10%)**	1.01 (1.01-1.02)^c^	1.01 (1.00-1.01)
**BMI (per 2** **kg/m**^**2**^**increase)**	1.04 (1.01-1.06)^c^	1.00 (0.97-1.02)
**History of cardiovascular disease**	1.09 (0.92-1.30)	1.19 (1.02-1.39)^c^
**Number of antihypertensive drugs classes prescribed (per increase of 1 drug class)**	0.78 (0.72-0.84)^c^	1.54 (1.45-1.64)^c^
**Number of visits to the PCP (reference: 0)**		
1 or 2	1.04 (0.93-1.15)	1.10 (1.00-1.22)^c^
3 or 4	1.07 (0.87-1.31)	1.21 (1.01-1.46)^c^
>4	1.11 (0.82-1.51)	1.34 (1.03-1.74)^c^
**Number of visits to nephrologist (reference: 0)**		
1 or 2	1.15 (1.02-1.30)^c^	1.16 (1.04-1.30)^c^
3 or 4	1.32 (1.04-1.68)^c^	1.35 (1.08-1.69)^c^
>4	1.52 (1.06-2.18)^c^	1.57 (1.12-2.19)^c^
**Number of visits to specialists in cardiology or diabetes (reference: 0)**		
1 or 2	1.25 (1.08-1.45)^c^	1.01 (0.88-1.16)
3 or 4	1.57 (1.17-2.11)^c^	1.02 (0.77-1.34)
>4	1.97 (1.27-3.07)^c^	1.02 (0.68-1.54)
**Legal status of the nephrology facility (reference: nonuniversity hospital)**		
University hospital	1.01 (0.84-1.22)	0.93 (0.74-1.17)
Private for-profit clinic	0.79 (0.61-1.02)	0.74 (0.53-1.04)
Private nonprofit clinic	1.62 (1.20-2.19)^c^	0.91 (0.58-1.44)

*Note:* For the adjusted model of withdrawals and of add-ons, the median frailty variance was 0.12 (95% CI 0.08-0.16, *P* < 0.001) and 0.00 (95% CI -0.04 to 0.04, *P* > 0.05), respectively.

Abbreviations: ACR, albumin-creatinine ratio; BMI, body mass index; BP, blood pressure; eGFR, estimated glomerular filtration rate; HR, hazard ratios; PCP, primary care physician.

a In the add-on model, the interaction term between systolic BP and the number of antihypertensive drug classes prescribed was significant. The HRs displayed for systolic BP were calculated for patients with 2 antihypertensive drug classes prescribed, whereas the that for the number of antihypertensive drug classes was calculated for patients with a systolic BP of 140 mm Hg (both medians values for these characteristics in the overall population).

b HRs of add-ons or withdrawals associated with systolic BP were derived with spline functions (Supplementary Figure S2). Comparisons against systolic BP at 120 mm Hg are based on exact systolic BP values (ie, 110, 130, 140, and 150 mm Hg).

c Indicates 95% confidence intervals excluding 1 (one).

After multivariable adjustment, the hazard of having an antihypertensive drug class withdrawn was higher among patients with any of the following: systolic BP <110 mm Hg, more antihypertensive drug classes at baseline, a CV history, more frequent visits to their PCP or nephrologist, or an education level of 9-11 versus ≥12 years. The hazard of withdrawal also varied significantly according to the patient's nephrologist (median frailty variance, 0.12; 95% CI, 0.08-0.16; *P* < 0.001).

The sensitivity analysis yielded associations similar to the main analysis (Supplementary Table S8) with no statistically significant association of the urinary sodium-creatinine ratio with add-on or withdrawal.

### Short-term Changes in Systolic BP Following Add-on of an Antihypertensive Drug Class

**Figure 3:**
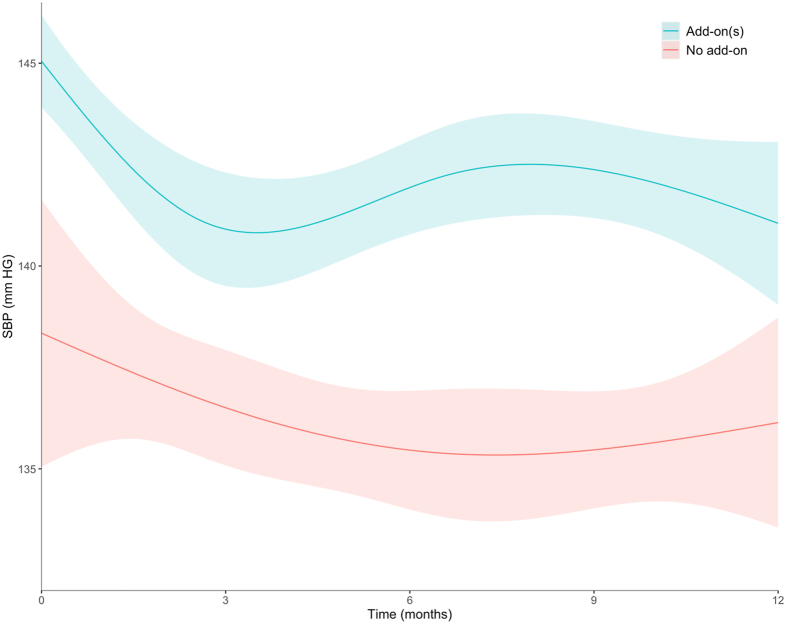
One-year systolic BP trends in patients with and without an add-on of an antihypertensive drug. In patients with add-on of an antihypertensive drug class, time zero corresponds to timing of add-on. In patients without add-on, time zero corresponds to 8.4 months from baseline (median time of the first add-on in patients who had it). Abbreviation: SBP, systolic blood pressure.

In patients with add-on of an antihypertensive drug class, mean systolic BP at start was 145 ± 1.1 mm Hg and was reduced by 4.1 mm Hg (95% CI, 2.1-6.2, Fig 3) in the first 3 months. After the third month, systolic BP increased slightly by 1.6 mm Hg (95% CI, −0.8 to 3.9), but without reaching its initial level up to 1 year after. In patients with no add-on, mean systolic BP was lower, 138 ± 3.3 mm Hg, and tended to decline smoothly, by 2.2 mm Hg (95% CI, −3.5 to 7.9), over the considered 1-year period.

## Discussion

In a large cohort of patients with moderate-to-severe CKD receiving care from a nephrologist, we identified substantial heterogeneity in antihypertensive prescriptions. Contrary to our initial hypothesis to explain the poor BP control in this population, we observed dynamic changes in these prescriptions over follow-up. The overall 20 per 100 patient-year rates for both add-ons and withdrawals nonetheless concealed disparities across drug classes, potentially informative about prescriber preferences and patient tolerance. We identified CV risk factors and comorbid conditions specifically associated with the add-on or withdrawal of drug classes. The higher hazards of add-on or withdrawal associated with more frequent physician visits seemed to vary by specialty. Importantly, our analysis showed that add-on of an antihypertensive drug class was associated with subsequent decrease in systolic BP level in CKD in real-world, yet this decrease was modest and poorly sustained in time.

We identified 257 distinct antihypertensive drug regimens at baseline (based on the type and number of drug classes). Over the study period, we observed that antihypertensive drug prescriptions were dynamic, with high rates of both drug class add-ons and withdrawals of 23 and 25 per 100 PY, respectively. This dynamic nonetheless resulted in a virtually stable distribution of the number of classes prescribed, but rates varied substantially by drug class. Our results indicate, for example, high rates of RASi prescription in patients not yet under RAS blockade. It is noteworthy that the MRA prescription rate was one-quarter that of centrally acting drugs (α-adrenergic antagonists and imidazoline). Despite MRA's well-established benefits in resistant hypertension and HF management,^26^^,^^27^ concerns about its life-threatening risks in CKD, including hyperkalemia and acute kidney injury, have precluded its widespread use in CKD patients.^28^

After adjustment for clinical and health care-related characteristics, patients with older age and higher BMI had higher rates of an antihypertensive drug add-on, consistent with the high prevalence of treatment-resistant hypertension in these populations.^29^ Moderate and poor adherence to drug therapy were common (55% and 7% of patients, respectively) and showed a graded association with a higher hazard of add-ons. This finding points to the need to evaluate adherence systematically before intensifying therapy. We also found nephrology care in private nonprofit clinics to be associated with greater likely of an add-on class compared with other types of facilities (public university or nonuniversity hospitals and private for-profit clinics). It is not clear whether this finding is because of specific care protocols or patient profiles because, in France, these clinics are often intended to care for patients with less complex CKD. On the other hand, the adjusted hazard of withdrawing an antihypertensive drug class was higher in patients with than without a history of CV disease. Deprescribing is part of the continuum of good prescription practices and may be particularly important among patients with multiple comorbid conditions and polypharmacy.^30^

Changes in these prescriptions were associated with more frequent medical visits in the preceding year. Except for nephrologists (similarly associated with add-ons and withdrawals), the associated physician specialties differed. The rate of add-ons was higher in patients seeing specialists (in cardiology, endocrinology, and diabetes) more often, whereas the rate of withdrawals was more likely in patients seeing their PCP more often. PCPs remain at the frontline of CKD management and may have to deal with medication-related complaints more frequently. With enhanced diagnosis and awareness of CKD, the number of patients with CKD has increased dramatically over the past 2 decades and demonstrated the need to develop optimal models of nephrology referral and coordinated care.^31^

Of note, the magnitude of decline in systolic BP decline (4.1 mm Hg) at 3 months in patients with an add-on was similar to that reported in a meta-analysis of randomized clinical trials of the effects of antihypertensive drugs on long-term BP at 6 months.^32^ However, systolic BP did not reach optimal levels and tended to rebound after 3 months from add-on, consistent with the rates of withdrawal in our study (as much that of add-on). This finding underlines the difficulty of achieving BP control in CKD in the real-world setting and calls for interventions to manage adverse drug reactions leading to drug withdrawal.

Our study has several strengths. It is based on a large number of patients with confirmed CKD diagnoses and hypertension recruited from a representative sample of nephrology outpatient facilities. Our detailed survey of prescribed drugs constitutes a unique aspect of this study that allowed us to identify all prescription changes over a 5-year follow-up and provide information for each antihypertensive drug class.

Our study also has several limitations. One-quarter of prescription or deprescription dates were imputed. Dosing and prescriber specialty could not be assessed. Lack of information about individual BP goals precluded formal assessment of therapeutic inertia. Finally, the study period (2013-2020) does not allow us to assess the adherence to the 2021 Kidney Disease: Improving Global Outcomes (KDIGO) guideline update for antihypertensive management and the impact of the introduction of new drugs. Our findings may, however, provide a basis for a future analysis of the trends in clinical practices and be useful for future clinical trials.

In conclusion, this study showed substantial heterogeneity and changes in antihypertensive prescription practices among patients with CKD, probably reflecting clinician preferences and the decreased tolerability of some drug classes. Our findings further suggest that specialists and PCPs have different roles in prescribing and deprescribing drugs for patients with CKD, which underlines the importance of coordinated care. The reduction in systolic BP following add-on of an antihypertensive drug class in the real-world seemed, however, modest, and not long-lasting, consistent with the high rates of withdrawal and frequent poor self-reported adherence in this population.

## Supplementary Material

Supplementary File (PDF) Figures S1-S2; Items S1-S2; Tables S1-S8.
