# Supplementary material for: Antihypertensive Treatment Patterns in CKD Stages 3 and 4: The CKD-REIN Cohort Study
Source: Kidney Med. 2024 Oct 9;6(12):100912. doi: 10.1016/j.xkme.2024.100912 (PMC11577237; doi:10.1016/j.xkme.2024.100912)
Supplement: Supplementary File (PDF) — Figures S1-S2; Items S1-S2; Tables S1-S8. [file mmc1.pdf]

(A) (B)

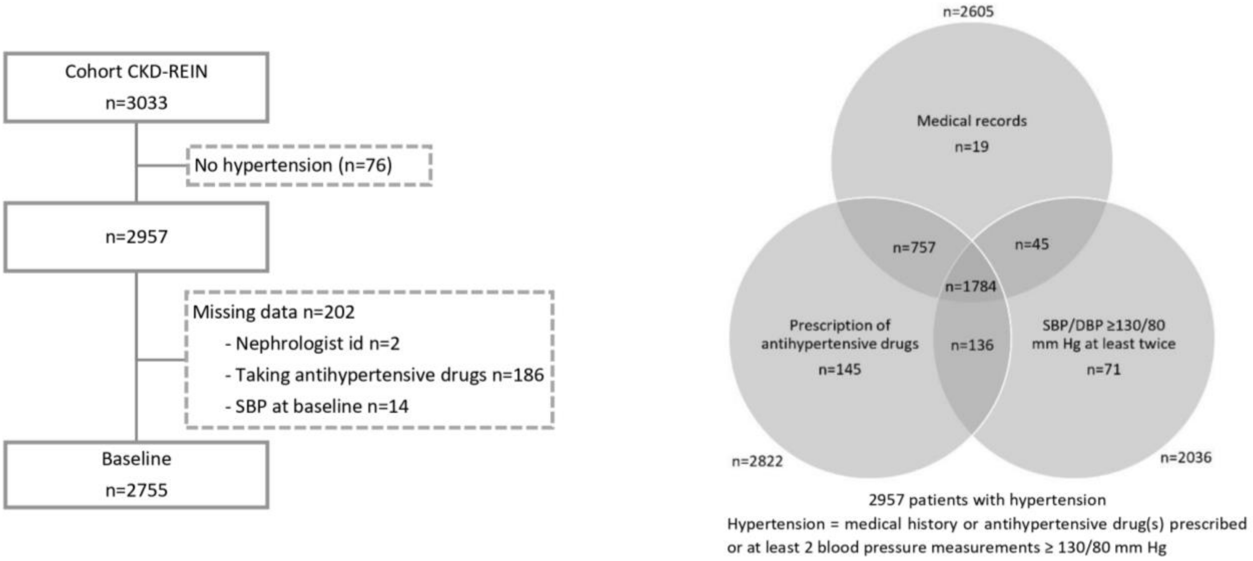

Figure S1. (A) Flow chart of selected patients at baseline in the CKD-REIN cohort and (B) Venn diagram of hypertension definition

Missing data on taking antihypertensive drugs either at baseline or on longitudinal follow-up.
Abbreviations: BP, blood pressure; CKD-REIN, Chronic Kidney Disease – Renal Epidemiology and Information Network; SBP, Systolic Blood Pressure; DBP, Diastolic Blood Pressure

Figure S2. Hazard Ratios and 95% confidence interval of natural cubic splines of the adjusted association between systolic blood pressure and (A) add-on, and (B) withdrawal hazard.

(A)

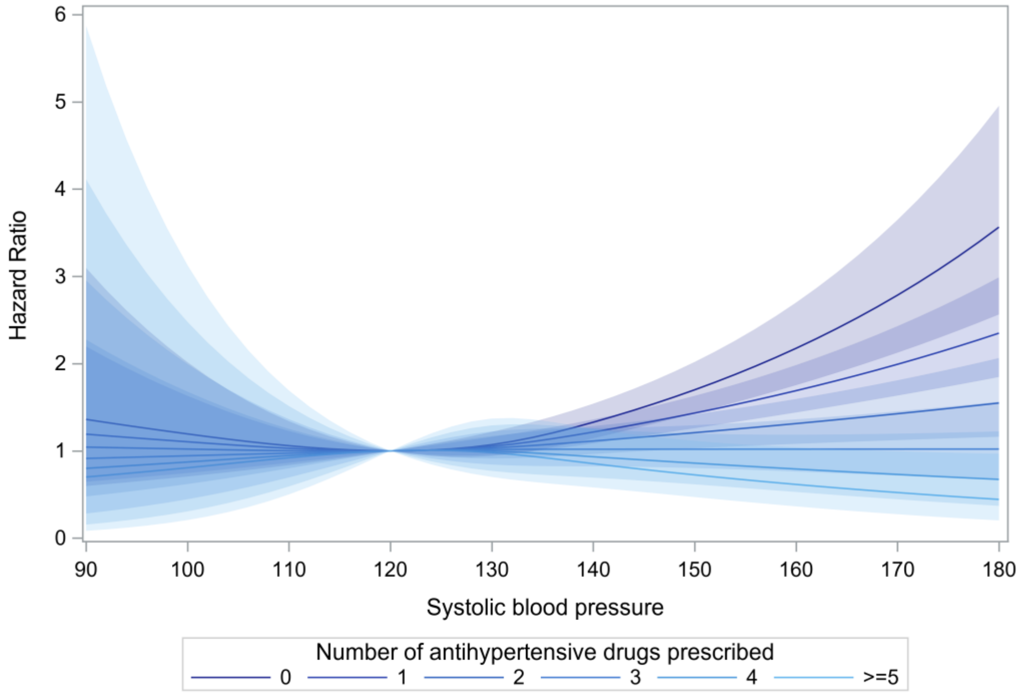

The interaction between systolic BP and the number of antihypertensive drug classes prescribed was considered

(B)

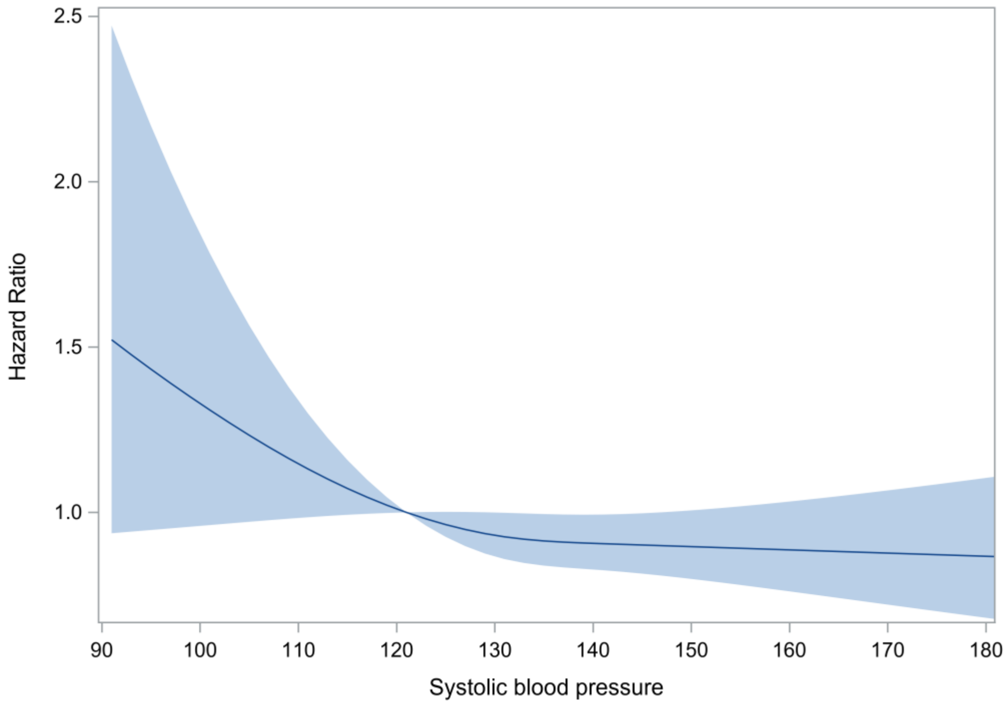

Table S1. Anatomical Therapeutic and Chemical (ATC) codes of antihypertensive drugs

Antihypertensive drugs	ATC codes
Thiazide diuretics	C02LA01 - C03AA03 - C03BA11 - C03BX03 - C03EA01- C07BB02 - C07BB07 - C07BB12 - C07CB03 - C07DA06 - C08GA02 - C09BA01 - C09BA02 - C09BA03 - C09BA04 - C09BA05 - C09BA06 - C09BA07 - C09BA09 - C09BA15 - C09BX01 - C09DA01 - C09DA03 - C09DA04 - C09DA06 - C09DA07 - C09DA08 - C09DX01 - C09EA04 - C09XA52
High-ceiling diuretics	C03CA01 - C03CA02 - C03CA03 - C03EB01
Mineralocorticoid receptor blockers	C03DA - C03EA04
Amiloride	C03DB01 - C03EB01 - C07DA06 - C03EA01
Beta-blockers	C07AA03 - C07AA05 - C07AA06 - C07AA12 - C07AA16 - C07AB02 - C07AB03 - C07AB04 - C07AB05 - C07AB07 - C07AB08 - C07AB12 - C07AG01 - C07BB02 - C07BB07 - C07BB12 - C07CB03 - C07DA06 - C07FB02 - C07FB03 - C09BX02
Calcium channel blockers	C08CA01 - C08CA02 - C08CA03 - C08CA04 - C08CA05 - C08CA08 - C08CA09 - C08CA11 - C08CA13 - C08DA01 - C08DB01 - C08GA02 - C07FB02 - C07FB03 - C09DX01 - C09BB02 - C09BB04 – C09BB07 - C09BB10 - C09DB01 - C09DB02 - C09DB04 - C09DB05 - C09BX01 - C10BX03 -
ACE inhibitors	C09AA01 - C09AA02 - C09AA03 - C09AA04 - C09AA05 - C09AA06 - C09AA07 - C09AA08 - C09AA09 - C09AA10 - C09AA15 - C09BB02 - C09BB04 - C09BB07 - C09BB10 - C09BA01 - C09BA02 - C09BA03 - C09BA04 - C09BA05 - C09BA06 - C09BA07 - C09BA09 - C09BA15 - C09BX01 - C09BX02
Angiotensin II antagonists	C09CA01 - C09CA02 - C09CA03 - C09CA04 - C09CA06 - C09CA07 - C09CA08 - C09DA01 - C09DA03 - C09DA04 - C09DA06 - C09DA07 - C09DA08 - C09DB01 - C09DB02 - C09DB04 - C09DB05 - C09DX01 – C09DX04
Renin-inhibitors	C09XA02 - C09XA52
Methyldopa	C02AB02
Imidazoline receptor agonists	C02AC01 - C02AC05 - C02AC06
Alpha-adrenoreceptor antagonists	C02CA01 - C02CA04 - C02CA06

Pyrimidine derivatives	C02DC01
Rauwolfia alkaloids	C02LA01

Table S2. Nephrology facility type and number of physician visits in the year preceding study enrollment, overall and by systolic blood pressure level (mm Hg).

Systolic blood pressure level<br>Characteristics	All (n=2755)	<120 (n=282)	120-129 (n=415)	130-139 (n=615)	140-159 (n=946)	≥ 160 (n=497)	Missing data n (%)
**Legal status of the nephrology facility, n (%)**							0 (0%)
Nonuniversity hospital	597 (22%)	52 (18%)	91 (22%)	126 (20%)	223 (24%)	105 (21%)	
University hospital	1,572 (57%)	192 (69%)	254 (61%)	341 (56%)	507 (53%)	278 (56%)	
Private for-profit clinic	421 (15%)	29 (10%)	53 (13%)	98 (16%)	153 (16%)	88 (18%)	
Private nonprofit clinic	165 (6%)	9 (3%)	17 (4%)	50 (8%)	63 (7%)	26 (5%)	
**Number of visits to the primary-care physician, n (%)**							345 (13%)
0	93 (4%)	5 (2%)	14 (4%)	22 (4%)	34 (4%)	18 (4%)	
1 or 2	304 (13%)	37 (15%)	55 (15%)	78 (15%)	94 (11%)	40 (9%)	
3 or 4	682 (28%)	66 (27%)	106 (28%)	163 (30%)	241 (29%)	106 (25%)	
More than 4	1,331 (55%)	133 (56%)	196 (53%)	274 (51%)	461 (56%)	267 (62%)	
**Number of visits to the nephrologist, n (%)**							345 (13%)
0	83 (3%)	14 (6%)	10 (3%)	18 (3%)	28 (3%)	13 (3%)	
1 or 2	1,496 (62%)	145 (60%)	248 (67%)	343 (65%)	508 (61%)	252 (58%)	
3 or 4	576 (24%)	57 (24%)	86 (23%)	120 (22%)	207 (26%)	106 (25%)	
More than 4	255 (11%)	25 (10%)	27 (7%)	56 (10%)	87 (10%)	60 (14%)	
**Number of visits to the specialist in cardiology or diabetes, n (%)**							345 (13%)
0	718 (30%)	79 (33%)	127 (35%)	175 (33%)	220 (26%)	117 (27%)	
1 or 2	1,414 (59%)	130 (54%)	209 (56%)	307 (57%)	506 (61%)	262 (61%)	
3 or 4	251 (10%)	29 (12%)	31 (8%)	47 (9%)	98 (12%)	46 (11%)	
More than 4	27 (1%)	3 (1%)	4 (1%)	8 (1%)	6 (1%)	6 (1%)	

**How do you judge the communication between your nephrologist and other doctors?, n (%)**							450 (16%)
Very good	775 (34%)	70 (30%)	111 (31%)	186 (36%)	276 (35%)	132 (33%)	
Good	1,228 (53%)	130 (56%)	205 (58%)	263 (50%)	422 (53%)	208 (52%)	
Fair	38 (2%)	4 (2%)	6 (2%)	13 (3%)	8 (1%)	7 (2%)	
Poor	8 (0%)	0 (0%)	2 (0%)	2 (0%)	2 (0%)	2 (0%)	
No opinion	256 (11%)	27 (12%)	32 (9%)	60 (11%)	86 (11%)	51 (13%)	
**Have you noticed any discrepancies in prescriptions or advice between your nephrologist and other doctors?, n (%)**							475 (17%)
Very often	17 (1%)	1 (0%)	4 (1%)	2 (0%)	5 (1%)	5 (1%)	
Often	104 (4%)	9 (4%)	15 (4%)	25 (5%)	34 (4%)	21 (5%)	
Rarely	409 (18%)	48 (21%)	58 (16%)	90 (18%)	137 (18%)	76 (19%)	
Never	1,293 (57%)	127 (56%)	214 (61%)	296 (57%)	454 (58%)	202 (51%)	
No opinion	457 (20%)	43 (19%)	64 (18%)	104 (20%)	152 (19%)	94 (24%)	

Table S3. Antihypertensive drug classes prescribed at baseline, by (A) SBP (B) CKD G-stage and (C) CKD A-stage.

(A)

**Systolic blood pressure levels**<br><br>**Antihypertensive drug classes prescribed**	**All (n=2755)**	**<120 (n=282)**	**120-129 (n=415)**	**130-139 (n=615)**	**140-159 (n=946)**	**≥ 160 (n=497)**
**RAS inhibitors, n (%)**	2,113 (77%)	231 (82%)	329 (79%)	472 (77%)	706 (75%)	375 (75%)
ACE inhibitors	943 (34%)	115 (41%)	151 (36%)	221 (36%)	302 (32%)	154 (31%)
Angiotensin receptor blockers	1,261 (46%)	125 (44%)	191 (46%)	271 (44%)	436 (46%)	238 (48%)
Direct renin inhibitors	29 (1%)	3 (1%)	2 (0%)	6 (1%)	13 (1%)	5 (1%)
**Diuretics, n (%)**	1,531 (56%)	135 (48%)	204 (49%)	313 (51%)	572 (60%)	307 (62%)
Thiazide and thiazide-like	561 (20%)	41 (15%)	71 (17%)	124 (20%)	223 (24%)	102 (21%)
High-ceiling (loop) diuretics	1,031 (37%)	98 (35%)	141 (34%)	195 (32%)	373 (39%)	224 (45%)
Mineralocorticoid receptor blockers	121 (4%)	20 (7%)	22 (5%)	27 (4%)	33 (3%)	19 (4%)
Amiloride	14 (1%)	0 (0%)	2 (0%)	4 (1%)	8 (1%)	0 (0%)
**Calcium channel blockers, n (%)**	1,308 (47%)	76 (27%)	139 (33%)	293 (48%)	499 (53%)	301 (61%)
**β-blocker, n (%)**	1,170 (42%)	126 (45%)	155 (37%)	232 (38%)	420 (44%)	237 (48%)
**Alpha-adrenergic antagonists, n (%)**	312 (11%)	11 (4%)	32 (8%)	56 (9%)	116 (12%)	97 (20%)
**Centrally acting drugs, n (%)**	199 (7%)	10 (4%)	15 (4%)	33 (5%)	77 (8%)	64 (13%)
Methyldopa	1 (0%)	0 (0%)	0 (0%)	0 (0%)	1 (0%)	0 (0%)
Imidazoline receptor agonists	198 (7%)	10 (4%)	15 (4%)	33 (5%)	76 (8%)	64 (13%)
Rauwolfia alkaloids (reserpine)	0 (0%)	0 (0%)	0 (0%)	0 (0%)	0 (0%)	0 (0%)
**Pyrimidine derivatives, n (%)**	1 (0%)	0 (0%)	0 (0%)	1 (0%)	0 (0%)	0 (0%)

(B)

**CKD G-stage**<br><br>**Antihypertensive drug classes**	**Stage 3A (n=480)**	**Stage 3B (n=1031)**	**Stage 4/5 (n=1244)**
**RAS inhibitors,** n (%)	381 (79%)	797 (77%)	935 (75%)
ACE inhibitors	169 (35%)	350 (34%)	424 (34%)

Angiotensin receptor blockers	224 (47%)	475 (46%)	562 (45%)
Direct renin inhibitors*	6 (1%)	8 (1%)	15 (1%)
**Diuretics,** n (%)	206 (43%)	549 (53%)	776 (62%)
Thiazide and thiazide-like	113 (24%)	231 (22%)	217 (17%)
High-ceiling (loop) diuretics	83 (17%)	345 (33%)	603 (48%)
Mineralocorticoid receptor blockers	32 (7%)	46 (4%)	43 (3%)
Amiloride	5 (1%)	4 (0%)	5 (0%)
**Calcium channel blockers,** n (%)	202 (42%)	461 (45%)	645 (52%)
**Beta-blocking agents,** n (%)	180 (38%)	415 (40%)	575 (46%)
**Alpha-adrenergic antagonists,** n (%)	45 (9%)	98 (10%)	169 (14%)
**Centrally acting drugs,** n (%)	23 (5%)	81 (8%)	95 (8%)
Methyldopa	0 (0%)	1 (0%)	0 (0%)
Imidazoline receptor agonists	23 (5%)	80 (8%)	95 (8%)
Rauwolfia alkaloids (reserpine)	0 (0%)	0 (0%)	0 (0%)
**Pyrimidine derivatives,** n (%)	0 (0%)	1 (0%)	0 (0%)

(C)

**CKD A-stage**<br><br>**Antihypertensive drug classes prescribed**	**Stage A1 (n=665)**	**Stage A2 (n=859)**	**Stage A3 (n=834)**
**RAS inhibitors,** n (%)	492 (74%)	662 (77%)	670 (80%)
ACE inhibitors	205 (31%)	289 (34%)	316 (38%)
Angiotensin receptor blockers	289 (43%)	396 (46%)	407 (49%)
Direct renin inhibitors	9 (1%)	5 (1%)	11 (1%)
**Diuretics,** n (%)	352 (53%)	467 (54%)	482 (58%)
Thiazide and thiazide-like	145 (22%)	155 (18%)	187 (22%)
High-ceiling (loop) diuretics	212 (32%)	329 (38%)	328 (39%)

Mineralocorticoid receptor blockers	48 (7%)	31 (4%)	26 (3%)
Amiloride	4 (1%)	4 (0%)	4 (0%)
**Calcium channel blockers,** n (%)	278 (42%)	391 (46%)	459 (55%)
**Beta-blocking agents,** n (%)	280 (42%)	348 (41%)	365 (44%)
**Alpha-adrenergic antagonists,** n (%)	47 (7%)	86 (10%)	127 (15%)
**Centrally acting drugs,** n (%)	42 (6%)	50 (6%)	73 (9%)
Methyldopa	0 (0%)	1 (0%)	0 (0%)
Imidazoline receptor agonists	42 (6%)	49 (6%)	73 (9%)
Rauwolfia alkaloids (reserpine)	0 (0%)	0 (0%)	0 (0%)
**Pyrimidine derivatives,** n (%)	0 (0%)	1 (0%)	0 (0%)

Chronic kidney disease G-stages are defined by the estimated glomerular filtration (eGFR) level or the need for kidney replacement therapy (KRT): 3A, 45≤eGFR<60 mL/min/1.73m²; 3B, 30≤eGFR<45 mL/min/1.73m²; 4, 15≤eGFR<30 mL/min/1.73m²; and 5, eGFR<15 mL/min/1.73m² or KRT. Chronic kidney disease A-stages are defined by the albumin creatinine ratio (ACR) level: A1, ACR<30 mg/g; A2, 30≤ACR<300 mg/g; and A3, ACR>300 mg/g.

Although direct renin inhibitors belong to the renin-angiotensin system inhibitor class, they are not recommended for first-line treatment of hypertension in CKD. They were withdrawn from the French market in October 2017.

Abbreviations: ACE, angiotensin converting enzyme; RAS, renin-angiotensin system

Table S4. Antihypertensive regimens* prescribed at baseline.

Regimens*	N (%)	Number of combinations*
**No antihypertensive drug prescribed**	135 (5%)	
**Only RASi**	405 (15%)	1
ACEi	171 (6%)	
ARBs	205 (7%)	
Other	29 (1%)	
**Diuretics and RASi**	246 (9%)	18
Thiazide(-like) and ARBs	69 (3%)	
Loop and ARBs	68 (2%)	
Other	109 (4%)	
**Diuretics, CCBs, and RASi**	241 (9%)	19
Loop, CCBs, and ARBs	67 (3%)	
Thiazide(-like), CCBs and ARBs	61 (2%)	
Other	113 (4%)	
**Diuretics, β-blockers, and RASi**	229 (8%)	20
Loop, β-blockers and ARBs	66 (2%)	
Loop, β-blockers and ACEi	65 (2%)	
Other	98 (4%)	
**Diuretics, β-blockers, CCBs, and RASi**	226 (8%)	17
Loop, β-blockers, CCBs and ARBs	62 (2%)	
Loop, β-blockers, CCBs and ACEi	51 (2%)	
Other	113 (4%)	
**CCBs and RASi**	178 (6%)	5
CCBs and ARBs	91 (3%)	
CCBs and ACE inhibitors	76 (3%)	
Other	11 (0.4%)	
**β-blockers and RASi**	127 (5%)	3
β-blockers and ARBs	61 (2%)	
β-blockers and ACEi	64 (2%)	
β-blockers, ACEi, and ARBs	2 (0.1%)	
**Diuretics, β-blockers, CCBs, RASi, and others**	115 (4%)	31
**Diuretics, CCBs, RASi, and others**	106 (4%)	26
**β-blockers, CCBs, and RASi**	104 (4%)	4
β-blockers, CCBs, and ACEi	54 (2%)	
β-blockers, CCBs, and ARBs	45 (2%)	
Other	5 (0.2%)	
**Diuretics and β-blockers**	85 (2.7%)	6
Loop and β-blockers	66 (2%)	
Other	19 (0.7%)	
**Only CCBs**	70 (3%)	0
**Only β-blockers**	60 (2%)	0
**Only Diuretics**	57 (2%)	3
Loop	44 (2%)	
Other	13 (0.5%)	
**Diuretics, β-blockers and CCBs**	55 (2%)	7
Loop, β-blockers and CCBs	42 (2%)	
Other	13 (0.5%)	
**Diuretics and CCBs**	45 (2%)	6
**Diuretics, β-blockers, CCBs, and others**	34 (1%)	11
**Diuretics, β-blockers, RASi, and others**	33 (1%)	18
**β-blockers, CCBs, RASi, and others**	32 (1%)	6
**β-blockers and CCBs**	31 (1%)	1
**CCBs, RASi, and others**	27 (1%)	10
**Diuretics, RASi, and others**	23 (0.8%)	10

**Diuretics, CCBs, and others**	19 (0.7%)	5
**β-blockers, CCBs, and others**	15 (0.5%)	3
**RASi and others**	13 (0.5%)	3
**Diuretics, β-blockers, and others**	12 (0.4%)	5
**CCBs and others**	10 (0.4%)	2
**β-blockers, RASi, and others**	8 (0.3%)	4
**Diuretics and others**	5 (0.2%)	2
**Only others**	5 (0.2%)	0
**β-blockers and others (α-blockers)**	4 (0.1%)	1
**Total**	2755 (100%)	**247**

Abbreviations: ACEi, angiotensin-converting enzyme inhibitors; ARBs, angiotensin II receptor blockers; CCBs, calcium channel blockers; Loop, High ceiling (loop) diuretics; RASi, renin-angiotensin system inhibitors; Thiazide(-like), Thiazide and thiazide-like diuretics.
*Regimens are defined by level 4 (chemical subgroup) of the Anatomic Therapeutic and Chemical (ATC) hierarchy.
Combinations correspond to at least 2 antihypertensive drugs prescribed. "Other" refers to regimens prescribed to fewer than 40 patients.

Table S5. Number of events, number of patients at risk, rates (95% confidence intervals) of (A) changes in antihypertensive drug class prescriptions overall, (B) add-ons, and (C) withdrawals by drug class.

(A) Changes in antihypertensive drug class prescriptions, overall

Type of changes	Changes (n)	Patients at risk (person-years)	Rate (95% CI, per 100 person-years)
Any changes	5265	10579	49.8 (48.4-51.1)
Withdrawals	2463	10030	24.6 (23.6-25.5)
Add-ons	2411	10579	22.8 (21.9-23.7)
Switches	391	10030	3.90 (3.51-4.28)

(B) Add-ons, by drug class

Antihypertensive drug prescriptions	Add-ons (n)	Patients at risk (person-years)	Rate (95% CI, per 100 person-years)
RASi	507	2654	19.1 (17.4-20.8)
ARBs*	313	5631	5.6 (4.9-6.2)
ACEi*	274	6863	3.99 (3.52-4.47)
CCBs	514	5345	9.6 (8.8-10.5)
Loop diuretics	443	6550	6.8 (6.1-7.4)
β-blockers	304	5767	5.3 (4.7-5.9)
Thiazide(-like) diuretics	277	8145	3.40 (3.00-3.80)
α-blockers	217	9045	2.40 (2.08-2.72)
Imidazoline	107	9438	1.13 (0.92-1.35)
Mineralocorticoid R-blockers	66	9727	0.68 (0.51-0.84)
Amiloride	11	10099	0.109 (0.045-0.173)
Methyldopa	2	10139	Non estimable*
Direct renin inhibitors	1	10086	Non estimable*

*80 add-ons are add-ons of a RASi to another RASi

(C) Withdrawals, by drug class

Antihypertensive drug prescriptions	Withdrawals (n)	Patients at risk (person-years)	Rate (95% CI, per 100 person-years)
Amiloride	12	45	26.8 (11.6-42.0)
Direct renin inhibitors	14	58	24.2 (11.5-36.9)
Mineralocorticoid R-blockers	92	416	22.1 (17.6-26.6)
Thiazide(-like) diuretics	315	1998	15.8 (14.0-17.5)
α-blockers	165	1098	15.0 (12.7-17.3)
Imidazoline	95	706	13.5 (10.8-16.2)
RASi	725	7186	10.1 (9.4-10.8)
ACEi**	367	3281	11.2 (10.0-12.3)
ARBs**	477	4513	10.6 (9.6-11.5)
Loop diuretics	353	3593	9.8 (8.8-10.9)
CCBs	449	4799	9.4 (8.49-10.22)
β-blockers	241	4377	5.5 (4.8-6.2)
Methyldopa	1	5	Non estimable*

*Non estimable with normal approximation

**119 withdrawals are withdrawals of a RAS inhibitor in patients with dual blockade.

Abbreviations: ACEi, angiotensin-converting enzyme inhibitors; ARBs, angiotensin II receptor blockers; CCBs, calcium channel blockers; Mineralocorticoid R-blockers, mineralocorticoid receptor blockers; RASi, renin-angiotensin system inhibitors; Thiazide(-like) diuretics, thiazide, and thiazide-like diuretics

Table S6. Antihypertensive drug classes prescribed at the time of changes, overall and by antihypertensive drug classes for (A) add-ons and (B) withdrawals.

(A)

**Add-ons**	**Add-ons**	**ACEi**	**ARBs**	**Thiazide (-like)**	**Loop diuretics**	**MRA**	**CCBs**	**β-blockers**	**α-blockers**	**Imidazoline**
Add-ons*, n	2,411	274	313	277	443	66	514	304	217	107
Patients with at least one add-on**, n	1,323	250	281	247	385	56	464	286	203	105
**Antihypertensive drug prescriptions**										
RASi, n (%)	1,174 (53%)	40 (15%)	34 (11%)	163 (59%)	279 (63%)	40 (61%)	330 (64%)	180 (59%)	133 (61%)	76 (71%)
ACEi	503 (23%)	**0 (0%)**	34 (11%)	73 (26%)	123 (28%)	21 (32%)	131 (25%)	73 (24%)	56 (26%)	32 (30%)
ARBs	723 (33%)	40 (15%)	**0 (0%)**	96 (35%)	172 (39%)	21 (32%)	210 (41%)	109 (36%)	89 (41%)	48 (45%)
Renin	17 (1%)	4 (1%)	3 (1%)	2 (1%)	2 (0%)	1 (2%)	4 (1%)	3 (1%)	0 (0%)	0 (0%)
Diuretics, n (%)	1,051 (48%)	138 (50%)	160 (51%)	91 (33%)	93 (21%)	48 (73%)	251 (49%)	168 (55%)	134 (62%)	77 (72%)
Thiazide(-like)	328 (15%)	39 (14%)	23 (7%)	**0 (0%)**	77 (17%)	11 (17%)	91 (18%)	47 (15%)	40 (18%)	30 (28%)
Loop	717 (32%)	101 (37%)	133 (42%)	85 (31%)	**0 (0%)**	40 (61%)	165 (32%)	120 (39%)	102 (47%)	49 (46%)
MRB	71 (3%)	4 (1%)	18 (6%)	11 (4%)	15 (3%)	**0 (0%)**	9 (2%)	6 (2%)	9 (4%)	5 (5%)
Amiloride	15 (1%)	1 (0%)	2 (1%)	1 (0%)	4 (1%)	1 (2%)	3 (1%)	2 (1%)	0 (0%)	1 (1%)
CCBs, n (%)	954 (43%)	113 (41%)	135 (43%)	155 (56%)	240 (54%)	40 (61%)	**0 (0%)**	140 (46%)	151 (70%)	78 (73%)
β-blockers, n (%)	925 (42%)	118 (43%)	146 (47%)	136 (49%)	217 (49%)	44 (67%)	209 (41%)	**0 (0%)**	104 (48%)	60 (56%)
α-blockers, n (%)	290 (13%)	29 (11%)	46 (15%)	43 (16%)	67 (15%)	17 (26%)	52 (10%)	38 (13%)	**0 (0%)**	29 (27%)
Centrally acting drugs, n (%)	171 (8%)	25 (9%)	28 (9%)	26 (9%)	43 (10%)	6 (9%)	24 (5%)	20 (7%)	29 (13%)	0 (0%)
Methyldopa	1 (0%)	0 (0%)	0 (0%)	1 (0%)	0 (0%)	0 (0%)	0 (0%)	0 (0%)	0 (0%)	0 (0%)
Imidazoline	170 (8%)	25 (9%)	28 (9%)	25 (9%)	43 (10%)	6 (9%)	24 (5%)	20 (7%)	29 (13%)	**0 (0%)**
**Number of antihypertensive drug classes prescribed, median (IQR)**	2 (1 – 3)	2 (1 – 3)	2 (1 – 3)	2 (1 – 3)	2 (1 – 3)	3 (2 – 4)	2 (1 – 2)	2 (1 – 2)	3 (2 – 4)	3 (2 – 4)

(B)

**Withdrawals**	**Withdrawals**	**ACEi**	**ARBs**	**Thiazide (-like)**	**Loop diuretics**	**MRA**	**CCBs**	**β-blockers**	**α-blockers**	**Imidazoline**
Withdrawals*, n	2,463	367	477	315	353	92	449	241	165	95
Patients with at least one withdrawal**, n	1,267	329	412	287	297	78	402	223	151	90
**Antihypertensive drug prescriptions**										
RASi, n (%)	1,705 (78%)	367 (100%)	477 (100%)	250 (79%)	229 (65%)	65 (71%)	331 (74%)	151 (63%)	111 (67%)	62 (65%)
ACEi	810 (37%)	**367 (100%)**	54 (11%)	108 (34%)	111 (31%)	29 (32%)	159 (35%)	76 (32%)	45 (27%)	25 (26%)
ARBs	1,045 (48%)	65 (18%)	**477 (100%)**	160 (51%)	130 (37%)	41 (45%)	199 (44%)	80 (33%)	76 (46%)	40 (42%)
Renin	28 (1%)	2 (1%)	1 (0%)	8 (3%)	1 (0%)	4 (4%)	6 (1%)	2 (1%)	0 (0%)	3 (3%)
Diuretics, n (%)	1,641 (75%)	223 (61%)	328 (69%)	315 (100%)	353 (100%)	92 (100%)	291 (65%)	175 (73%)	120 (73%)	80 (84%)
Thiazide(-like)	602 (28%)	83 (23%)	114 (24%)	**315 (100%)**	61 (17%)	16 (17%)	101 (22%)	53 (22%)	29 (18%)	29 (31%)
Loop	1,210 (56%)	160 (44%)	249 (52%)	116 (37%)	**353 (100%)**	55 (60%)	215 (48%)	132 (55%)	98 (59%)	64 (67%)
MRB	171 (8%)	11 (3%)	16 (3%)	15 (5%)	19 (5%)	**92 (100%)**	20 (4%)	14 (6%)	9 (5%)	10 (11%)
Amiloride	22 (1%)	2 (1%)	4 (1%)	5 (2%)	1 (0%)	1 (1%)	2 (0%)	1 (0%)	1 (1%)	0 (0%)
CCBs, n (%)	1,389 (64%)	198 (54%)	263 (55%)	188 (60%)	196 (56%)	52 (57%)	**449 (100%)**	130 (54%)	123 (75%)	75 (79%)
β-blockers, n (%)	1,211 (56%)	171 (47%)	249 (52%)	149 (47%)	195 (55%)	52 (57%)	219 (49%)	**241 (100%)**	97 (59%)	57 (60%)
α-blockers, n (%)	443 (20%)	43 (12%)	82 (17%)	55 (17%)	47 (13%)	21 (23%)	64 (14%)	28 (12%)	**165 (100%)**	31 (33%)
Centrally acting drugs, n (%)	280 (13%)	33 (9%)	49 (10%)	31 (10%)	41 (12%)	5 (5%)	45 (10%)	22 (9%)	29 (18%)	95 (100%)
Methyldopa	1 (0%)	0 (0%)	0 (0%)	0 (0%)	0 (0%)	0 (0%)	0 (0%)	0 (0%)	0 (0%)	0 (0%)
Imidazoline	279 (13%)	33 (9%)	49 (10%)	31 (10%)	41 (12%)	5 (5%)	45 (10%)	22 (9%)	29 (18%)	**95 (100%)**
**Number of antihypertensive drugs prescribed, median (IQR)**	3 (2 - 4)	3 (2 - 4)	3 (2 - 4)	3 (3 - 4)	3 (2 - 4)	4 (3 - 5)	3 (2 - 4)	3 (2 - 4)	4 (3 - 5)	5 (4 - 5)

*Number of all changes - either add-ons or withdrawals - over the 5-year follow-up
**Patients can have multiples changes - either add-ons or withdrawals - of the same antihypertensive drug class, over the 5-year follow-up.
Tables display antihypertensive drug prescriptions at the time of change by drug classes with at least 50 events.
Abbreviations: ACEi, angiotensin-converting enzyme inhibitors; ARBs, angiotensin II receptor blockers; CCBs, calcium channel blockers; MRA, mineralocorticoid receptor antagonists; RASi, renin-angiotensin system inhibitors; Thiazide(-like) diuretics, thiazide, and thiazide-like diuretics

Table S7. Crude hazard ratios of add-on and withdrawal of an antihypertensive drug class associated with patient- and provider-related factors.

Factors	HR (95% CI) Crude models	
	Add-ons	Withdrawals
Systolic BP **(**mm Hg, reference: 120 mm Hg**)**		
110 mm Hg	0.96 (0.81-1.15)	1.14 (0.99-1.31)
130 mm Hg	1.09 (0.99-1.19)	0.96 (0.89-1.04)
140 mm Hg	**1.24 (1.11-1.39)**	1.04 (0.95-1.13)
150 mm Hg	**1.44 (1.28-1.63)**	**1.14 (1.02-1.27**)
Age (per 10-year increase)	**1.08 (1.02-1.15)**	**1.12 (1.05-1.18)**
Men	0.95 (0.82-1.11)	1.11 (0.95-1.29)
Education (years, reference: ≥ 12)		
9-11	**1.27 (1.01-1.59)**	**1.54 (1.26-1.89)**
< 9 years	1.18 (1.00-1.39)	1.08 (0.93-1.27)
Adherence (reference: good)		
Moderate	1.10 (0.94-1.28)	**1.40 (1.20-1.64)**
Poor	1.31 (0.99-1.74)	1.23 (0.92-1.66)
Diabetes	1.16 (1.00-1.35)	**1.45 (1.26-1.68)**
eGFR (per decrease of 10 mL/min/1.73m²)	**1.07 (1.01-1.14)**	**1.19 (1.12-1.27)**
ACR (mg/g, per increase of 10%)	**1.01 (1.01-1.02)**	**1.01 (1.01-1.02)**
BMI (per 2 kg/m² increase)	1.03 (1.00-1.05)	**1.06 (1.04-1.09)**
History of cardiovascular disease	1.12 (0.96-1.30)	**1.57 (1.37-1.81**)
Number of antihypertensive drugs classes prescribed (per increase of 1 drug class)	**0.85 (0.81-0.91)**	**1.61 (1.52-1.70)**
Number of visits to the primary-care physician (reference: 0)		
1 or 2	1.09 (0.99-1.20)	**1.20 (1.08-1.32)**
3 or 4	1.18 (0.97-1.43)	**1.43 (1.17-1.75)**
More than 4	1.28 (0.96-1.71)	**1.71 (1.27-2.31)**
Number of visits to nephrologist (reference: 0)		
1 or 2	**1.23 (1.10-1.38)**	**1.28 (1.15-1.42)**
3 or 4	**1.52 (1.22-1.89)**	**1.64 (1.33-2.02)**
More than 4	**1.87 (1.34-2.61)**	**2.10 (1.53-2.87)**
Number of visits to specialist in cardiology or diabetes (reference: 0)		
1 or 2	**1.24 (1.10-1.40)**	**1.30 (1.16-1.46)**
3 or 4	**1.54 (1.21-1.96)**	**1.68 (1.34-2.12)**
More than 4	**1.90 (1.32-2.73)**	**2.18 (1.54-3.08)**
Legal status of the nephrology facility (reference: non university hospital)		
University hospital	0.96 (0.80-1.16)	0.86 (0.68-1.09)
Private for-profit clinic	0.83 (0.65-1.07)	0.69 (0.49-0.98)
Private nonprofit clinic	1.33 (0.99-1.79)	0.76 (0.48-1.20)

Abbreviations: ACR, albumin creatinine ratio; BMI, body mass index; BP, blood pressure; eGFR, estimated glomerular filtration rate; IQR, interquartile range; KDIGO, Kidney Disease Improving Global Outcomes; SD, standard deviation

Table S8. Sensitivity analysis**:** Crude and adjusted hazard ratios of add-ons and withdrawals of antihypertensive drug classes associated patient- and provider-related factors with complete data for sodium-to-creatinine ratio (n=1975).

**Factors**	**HR (95% CI) Add-ons**		**HR (95% CI) Withdrawals**	
	**Crude Model**	**Adjusted Model#**	**Crude Model**	**Adjusted Model**
Systolic BP (mm Hg, reference: 120 mm Hg*)				
110 mm Hg	0.94 (0.77-1.15)	0.98 (0.76-1.26)	1.16 (0.99-1.36)	1.17 (1.00-1.36)
130 mm Hg	1.09 (0.98-1.22)	1.03 (0.89-1.19)	0.95 (0.88-1.04)	**0.91 (0.84-0.99)**
140 mm Hg	**1.23 (1.09-1.40)**	1.07 (0.91-1.27)	1.02 (0.92-1.13)	**0.88 (0.80-0.98)**
150 mm Hg	**1.40 (1.21-1.61)**	1.11 (0.92-1.35)	1.12 (0.99-1.27)	**0.87 (0.76-0.99)**
Age (per 10-year increase)	**1.10 (1.03-1.18)**	**1.13 (1.03-1.23)**	**1.12 (1.04-1.20)**	1.07 (0.98-1.16)
Men	0.93 (0.77-1.11)	0.92 (0.76-1.12)	1.11 (0.93-1.32)	0.96 (0.80-1.16)
Education (years, reference: ≥ 12)				
9-11	**1.35 (1.03-1.77)**	1.18 (0.88-1.57)	**1.58 (1.24-2.02)**	**1.33 (1.03-1.73)**
< 9	1.17 (0.97-1.42)	1.04 (0.85-1.27)	1.18 (0.98-1.41)	1.04 (0.86-1.26)
Adherence (reference: good)				
Moderate	1.10 (0.91-1.32)	1.13 (0.94-1.36)	**1.38 (1.16-1.65)**	1.11 (0.93-1.34)
Poor	1.36 (0.96-1.95)	1.29 (0.89-1.86)	1.39 (0.97-1.98)	1.04 (0.72-1.49)
Diabetes	1.14 (0.96-1.36)	0.91 (0.74-1.13)	**1.42 (1.20-1.67)**	0.88 (0.72-1.07)
eGFR (per decrease of 10 mL/min/1.73m²)	**1.10 (1.02-1.19)**	1.03 (0.94-1.11)	**1.18 (1.10-1.27)**	1.02 (0.95-1.11)
ACR (mg/g, per increase of 10%)	**1.01 (1.01-1.02)**	**1.02 (1.01-1.02)**	**1.01 (1.01-1.02)**	1.01 (1.00-1.01)
BMI (per increase of 2 kg/m²)	1.03 (1.00-1.06)	1.04 (1.00-1.07)	**1.06 (1.03-1.09)**	0.99 (0.96-1.03)
Urinary Sodium-to-Creatinine Ratio (per increase of 1)	1.01 (1.00-1.02)	1.00 (0.99-1.01)	1.01 (1.00-1.02)	1.00 (0.99-1.01)
History of cardiovascular disease	1.18 (0.98-1.42)	1.13 (0.92-1.40)	**1.66 (1.40-1.96)**	**1.25 (1.04-1.51)**
Number of antihypertensive drugs classes prescribed (per increase of 1 drug class)	**0.85 (0.80-0.91)**	**0.78 (0.71-0.85)****	**1.59 (1.49-1.70)**	**1.53 (1.43-1.65)**
Number of visits to the primary-care physician (reference: 0)				
1 or 2	1.13 (1.00-1.27)	1.07 (0.94-1.21)	**1.21 (1.08-1.36)**	1.11 (0.99-1.25)
3 or 4	**1.27 (1.01-1.61)**	1.14 (0.89-1.46)	**1.46 (1.16-1.85)**	1.24 (0.97-1.57)

More than 4	**1.44 (1.01-2.04)**	1.22 (0.84-1.76)	**1.77 (1.25-2.52)**	1.37 (0.96-1.97)
Number of visits to nephrologist (reference: 0)				
1 or 2	**1.23 (1.08-1.40)**	1.13 (0.98-1.30)	**1.23 (1.09-1.40)**	1.12 (0.98-1.28)
3 or 4	**1.52 (1.18-1.96)**	1.27 (0.96-1.68)	**1.52 (1.19-1.96)**	1.25 (0.95-1.63)
More than 4	**1.87 (1.28-2.75)**	1.43 (0.94-2.18)	**1.88 (1.29-2.74)**	1.39 (0.93-2.09)
Number of visits to specialist in cardiology or diabetes (reference: 0)				
1 or 2	**1.28 (1.11-1.47)**	**1.28 (1.07-1.52)**	**1.30 (1.14-1.48)**	1.01 (0.86-1.17)
3 or 4	**1.63 (1.23-2.16)**	**1.63 (1.16-2.31)**	**1.70 (1.31-2.20)**	1.01 (0.74-1.38)
More than 4	**2.08 (1.36-3.18)**	**2.09 (1.24-3.50)**	**2.21 (1.50-3.26)**	1.02 (0.64-1.61)
Legal status of the nephrology facility (reference: nonuniversity hospital)				
University hospital	0.95 (0.75-1.19)	1.01 (0.80-1.26)	0.87 (0.68-1.13)	0.96 (0.74-1.23)
Private for-profit clinic	0.74 (0.53-1.03)	0.73 (0.52-1.02)	0.70 (0.47-1.03)	0.79 (0.53-1.16)
Private nonprofit clinic	1.22 (0.85-1.74)	**1.57 (1.10-2.25)**	0.74 (0.45-1.21)	0.89 (0.55-1.44)

For the adjusted models of withdrawals and of add-ons, the median frailty variance was 0.11, 95% CI 0.03-0.20, *P*< 0.01 and 0.01, 95% CI -0.05 to 0.07, *P*>0.05 respectively.

Abbreviations: ACR, albumin creatinine ratio; BMI, body mass index; BP, blood pressure; eGFR, estimated glomerular filtration rate.

* HRs of add-ons or withdrawals associated with SBP were derived with spline functions. Comparisons against SBP at 120 mm Hg are based on exact SBP values (i.e., 110, 130, 140, and 150 mm Hg).

#In the add-on model, the interaction term between systolic BP and the number of antihypertensive drug classes prescribed was significant. The HRs displayed for systolic BP were calculated for patients with 2 antihypertensive drug classes prescribed, while the that for the number of antihypertensive drug classes was calculated for patients with a systolic BP of 140 mm Hg (both medians values for these characteristics in the overall population).

Item S1: Supplementary methods

In the frailty model, fixed effects were age, sex, years of formal education, adherence to medication, BMI, eGFR, diabetes, SBP (natural cubic spline with knots at 100, 130, and 140 mm Hg), history of CV disease (including HF, CAD, and CVD), number of antihypertensive drugs prescribed, number of visits with PCP, nephrologist, and endocrinologist or cardiologist, log of ACR, and legal status of the nephrology facility (nonuniversity hospital, university hospital, private for-profit clinic, or private nonprofit clinic).

Item S2 : **CKD-REIN clinical sites and investigators**

Alsace: Prs T. Hannedouche et B. Moulin (CHU, Strasbourg), Dr A. Klein (CH Colmar) *Aquitaine*: Pr C. Combe (CHU, Bordeaux), Dr J.P. Bourdenx (Clinique St Augustin, Bordeaux), Dr A. Keller, Dr C. Delclaux (CH, Libourne), Dr B. Vendrely (Clinique St Martin, Pessac), Dr B. Deroure (Clinique Delay, Bayonne), Dr A. Lacraz (CH, Bayonne) *Basse Normandie*: Dr T. Lobbedez (CHU, Caen), Dr I. Landru (CH, Lisieux) *Ile de France*: Pr Z. Massy (CHU, Boulogne – Billancourt), Pr P. Lang (CHU, Créteil), Dr X. Belenfant (CH, Montreuil), Pr E. Thervet (CHU, Paris), Dr P. Urena (Clinique du Landy, St Ouen), Dr M. Delahousse (Hôpital Foch, Suresnes) *Languedoc – Roussillon*: Dr C. Vela (CH, Perpignan) *Limousin* : Pr M. Essig, Dr D. Clément (CHU, Limoges) *Lorraine*: Dr H. Sekhri, Dr M. Smati (CH, Epinal) Dr M. Jamali, Dr B. Hacq (Clinique Louis Pasteur, Essey-les-Nancy), Dr V. Panescu, Dr M. Bellou (Polyclinique de Gentilly, Nancy), Pr Luc Frimat (CHU, Vandœuvre-les-Nancy) *Midi-Pyrénées*: Pr N Kamar (CHU, Toulouse) *Nord-Pas-de-Calais*: Prs C. Noël et F. Glowacki (CHU, Lille), Dr N. Maisonneuve (CH, Valenciennes), Dr R. Azar (CH, Dunkerque), Dr M. Hoffmann (Hôpital privé La Louvière, Lille) *Pays-de-la Loire*: Pr M. Hourmant (CHU, Nantes), Dr A. Testa (Centre de dialyse, Rezé), Dr D. Besnier (CH, St Nazaire) *Picardie*: Pr G. Choukroun (CHU, Amiens), Dr G. Lambrey (CH, Beauvais) *Provence-Alpes - Côte d'Azur*: Pr S. Burtey (CHU, Marseille), Dr G. Lebrun (CH, Aix-en-Provence), Dr E. Magnant (Polyclinique du Parc Rambot, Aix-en-Provence) *Rhône-Alpes*: Pr M. Laville, Pr D. Fouque (CHU, Lyon-Sud) et L. Juillard (CHU Edouard Herriot, Lyon), Dr C. Chazot (Centre de rein artificiel Tassin Charcot, Ste Foy-les-Lyon), Pr P. Zaoui (CHU, Grenoble), Dr F. Kuentz (Centre de santé rénale, Grenoble).
